# Diagnostic accuracy of screening tools for silent aspiration in patients with dysphagia: a systematic review and meta-analysis

**DOI:** 10.3389/fneur.2025.1576869

**Published:** 2025-09-10

**Authors:** Wen-Jing Sun, Wen-Yao Cui, Yan Jiang, Wen-Jie Liu

**Affiliations:** ^1^Department of Neurosurgery, West China Hospital, Sichuan University, Chengdu, China; ^2^West China School of Nursing, Sichuan University, Chengdu, China; ^3^Nursing Department, West China Hospital, Sichuan University, Chengdu, China

**Keywords:** silent aspiration, dysphagia, diagnostic accuracy, systematic review, meta-analysis

## Abstract

**Background:**

The diagnosis and screening of silent aspiration are crucial for patients with dysphagia. This study aimed to synthesize the evidence and evaluate the diagnostic accuracy of screening tools for silent aspiration in patients with dysphagia.

**Methods:**

A comprehensive search of 6 databases including Pubmed, Web of Science, CINAHL, Cochrane Library, Scopus, and Embase was conducted from database inception to July 1st, 2024. Meta-analysis was performed on more than three studies. The bivariate mixed effect model was used to pool the sensitivity, specificity, positive likelihood ratio (PLR), negative likelihood ratio (NLR), and diagnostic odds ratio (DOR). Narrative analysis was applied for studies that could not conduct meta-analysis.

**Results:**

A total of nine studies were identified, which included six screening tools. Five of these screening tools for silent aspiration were analyzed descriptively. The meta-analysis was conducted to calculate the diagnostic accuracy of cough reflex test (CRT). The combined sensitivity and specificity of CRT were 0.65 (95% CI: 0.38–0.85) and 0.71 (95% CI: 0.63–0.79), respectively. The PLR, NLR, and DOR were 2.27 (95% CI: 1.49–3.47), 0.49 (95% CI: 0.24–0.99), and 4.68 (95% CI: 1.57–13.98), respectively. The area under the SROC curve was 0.73 (95% CI: 0.69–0.77).

**Conclusion:**

The videofluoroscopic swallowing study (VFSS) and flexible endoscopic evaluation of swallowing (FEES) remain the widely used gold standards for diagnosing silent aspiration. The CRT demonstrates moderate value in diagnosing and predicting silent aspiration. Further studies are needed to compare the diagnostic accuracy and predictive value of the remaining five screening tools for silent aspiration.

**Systematic review registration:**

Identifier CRD42023493439.

## 1 Introduction

Dysphagia is a common clinical symptom resulting from nervous system diseases, as well as oropharyngeal and esophageal diseases. Oropharyngeal dysphagia is closely related to aging, neurological diseases (such as Parkinson’s disease), dementia, head and neck cancer and other factors. The prevalence of dysphagia in the general population ranges from 7.3 to 29.6%, but it is as high as 72.4% among people with dementia (1). It is estimated that one in 25 adults suffers from dysphagia in America (2). The incidence and prevalence of dysphagia increase with age. In nursing home residents aged 60 and above, the incidence of dysphagia is 31.1%, leading to complications such as aspiration, malnutrition, and pneumonia (3). Currently, several diseases such as stroke, neurodegenerative diseases, dementia, Parkinson’s disease, and amyotrophic lateral sclerosis have been proven to be highly associated with dysphagia and aspiration events (4).

Impaired swallowing function, along with reduced muscle strength and coordination in the throat, increases the likelihood of food or liquid inadvertently entering the airway, leading to a significant rise in the incidence of aspiration (5). Aspiration can be divided into overt and silent aspiration. Silent aspiration, first described in 1937, is a process by which food residues, stomach contents, and oral secretions enter the airway or lungs below the glottis without coughing (6). Silent aspiration is usually associated with damage to the central or local muscle tissue of the pharynx, decreased sensation in the throat, and diminished cough reflex (7). As an invisible killer, the incidence of pneumonia caused by silent aspiration is much higher than that of overt aspiration. In acute hospitals, 58.2% of aspiration pneumonia cases are caused by silent aspiration (8). According to a survey, the mortality rate of patients with silent aspiration is 2.65 times that of patients without silent aspiration (8). In light of the high mortality and prevalence rate, the precise diagnosis of silent aspiration is essential for patients with dysphagia.

To date, various diagnostic tools for aspiration have been developed. However, screening and identifying silent aspiration becomes difficult due to the lack of a cough reflex. Silent aspiration is an easily missed and misdiagnosed disease because the initial symptoms and signs are not obvious. Instrumental examination is the gold standard for silent aspiration diagnosis, including videofluoroscopic swallowing study (VFSS) and flexible endoscopic evaluation of swallowing (FEES) (9). Due to the high costs, complex procedures, and the need for specialized trained staff, VFSS and FEES are not always practical (10). In addition to the instrumental examination, some clinical evaluation tools have also been developed to screen silent aspiration, such as Cough Reflex Tests, Pulse Oximetry Monitoring, and Clinical Swallow Examination (11–13). Several diagnostic trials have compared the accuracy of these screening tools, but there has been no systematic review to integrate these tools effectively.

Thus, this study used FEES or VFSS as the gold standard and performed a diagnostic meta-analysis to explore the accuracy of different screening and diagnostic tools for silent aspiration to better guide clinical application.

## 2 Materials and methods

This systematic review was reported according to the Preferred Reporting Items for Systematic Review and Meta-Analysis of Diagnostic Test Accuracy Studies (PRISMA-DTA) checklist and registered in PROSPERO.

### 2.1 Search strategy

We comprehensively searched 6 electronic databases, including Pubmed, Web of Science, CINAHL, Cochrane Library, Scopus, and Embase. The final search was conducted on July 1st, 2024. Only studies published in English were eligible. Keywords and MeSH terms were combined to search the databases. The complete search strategies are listed in Supplementary Table 1.

### 2.2 Inclusion and exclusion criteria

The included studies should meet the following eligible criteria: (1) patients with dysphagia aged more than 18 years old; (2) diagnostic studies comparing at least two silent aspiration screening tools; (3) FEES or VFSS as gold standard; (4) Outcomes including false negative value (FN), false positive value (FP), true negative value (TN), and true positive value (TP) could be obtained directly or indirectly; and (5) written in English. The exclusion criteria were as follows: (1) reviews, posters, meeting abstracts, and protocols, letters; (2) studies where silent aspiration cannot be distinguished; (3) animal research; and (4) studies lacking outcome data.

### 2.3 Data extraction

Two trained investigators independently screened, reviewed, and extracted the literature. If there was disagreement, a third reviewer decided whether to include the study. Data extraction included the first author, country, year of publication, sample size, age, disease, screening tools, gold standard, the incidence of silent aspiration (gold standard), the ratio of silent aspiration to aspiration (gold standard), TP, FP, TN, and FN values. In addition, the methodologies of 8 cough reflex tests (CRTs) were extracted based on the name of CRTs, induction substance, induction time, method of application, and results interpretation.

### 2.4 Risk of bias assessment

The Quality Assessment of Diagnostic Accuracy Studies (QUADAS-2) was used to assess the quality by two reviewers. Any disagreements were resolved by a third reviewer. The QUADAS-2 included two domains: risk of bias and clinical applicability evaluation. The risk of bias consists of four parts: patient selection, index test, reference standard, and flow and timing. The first three parts were also assessed in clinical applicability. Each part was assessed as high, medium, or low quality.

### 2.5 Statistical analysis

A meta-analysis was conducted whenever more than 3 studies with sufficient information were available. For studies that could not conduct meta-analysis, we calculated the sensitivity and specificity and performed a narrative analysis by Review Manager 5.3 software. Stata 12.0 was applied to calculate Spearman’s correlation coefficient to determine whether there was a threshold effect. The negative Spearman’s correlation coefficient indicated that there was no heterogeneity caused by threshold effects. Thus, the bivariate mixed effect model was used to pool the sensitivity, specificity, positive likelihood ratio (PLR), negative likelihood ratio (NLR), and diagnostic odds ratio (DOR). The PLR is the ratio of the true positive rate to the false positive rate. The NLR is the ratio of false negative rate to true negative rate. The DOR indicates the ability of diagnostic tests to distinguish patients from non-patients. The Symmetric Receiver Operating Curve (SROC) was drawn to analyze the diagnostic effect of CRT on silent aspiration. The closer the area under the SROC curve (AUC) is to 1, the better the diagnostic value (14). The Deeks’ funnel plot was used to assess publication bias, and Fagan’s nomogram was used to evaluate clinical utility. The Meta-diSc 1.4 software was used for subgroup analysis. The subgroups were defined by sample size, gold standard, induction substance, and induction time. Sensitivity analysis was carried out using the one-by-one elimination method to test the stability of the results.

## 3 Results

### 3.1 Study selection

**Figure 1:**
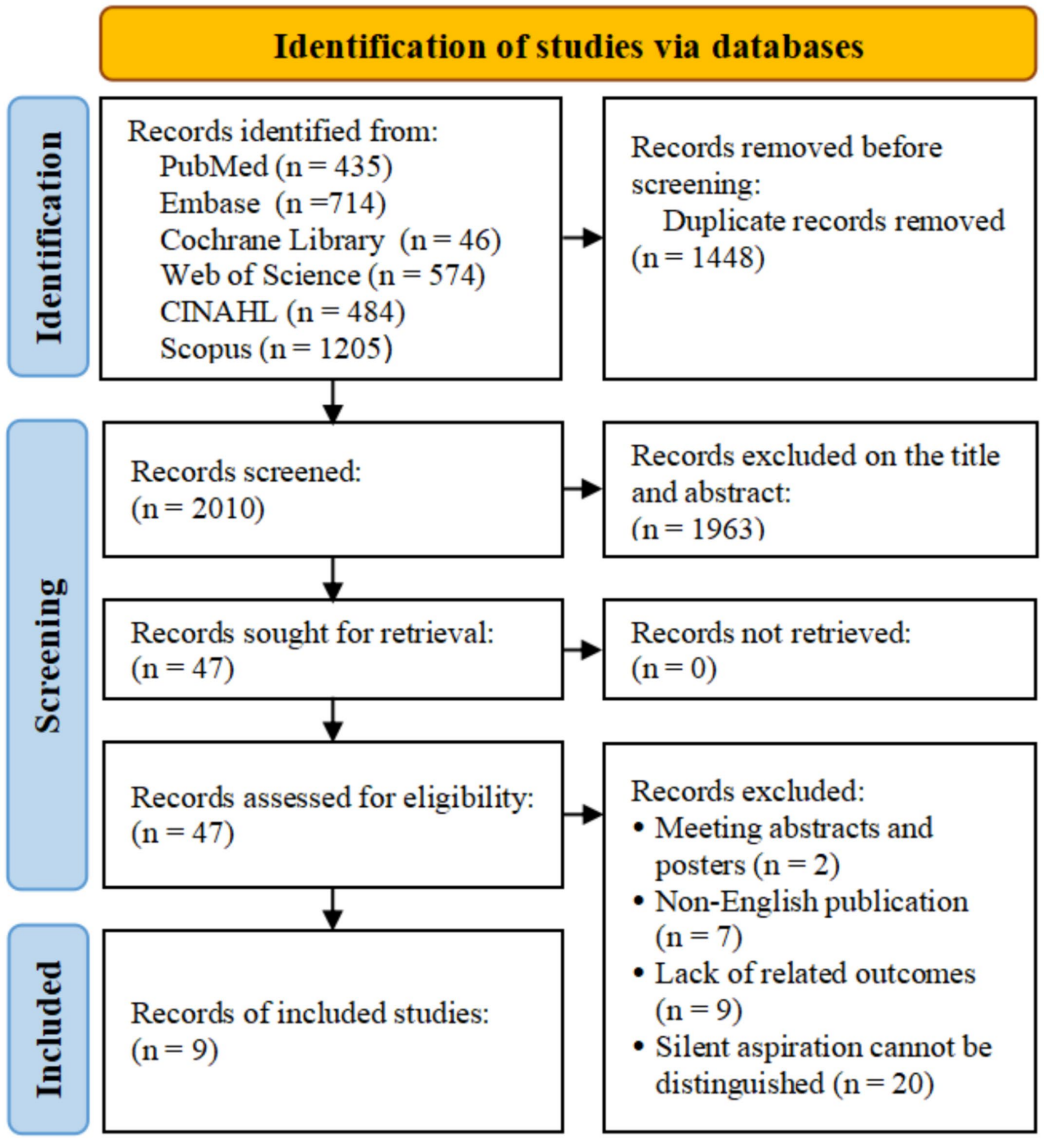
Flow diagram of study selection.

Through electronic retrieval, we initially identified 3,458 related studies and excluded 1,448 duplicate studies. There were 2,010 studies excluded from the titles and abstracts. After reading the full text, we excluded 2 meeting abstracts and posters, 7 non-English studies, and 9 studies lacking relevant outcomes. Twenty studies were excluded due to the difficulty in distinguishing overt and silent aspiration. Finally, 9 studies were included in this study (11–13, 15–20). The flow diagram of study selection is shown in Figure 1.

### 3.2 Study characteristics

This study included 920 patients with dysphagia from 6 counties. Three studies involved more than two screening tools for silent aspiration and were therefore analyzed independently (12, 13, 16). Only one study used FEES as the gold standard, 2 studies used FEES or VFSS as the gold standard, and others used VFSS as the gold standard. Six screening tools were applied to analyze the diagnostic accuracy, including the Modified Bedside Swallowing Assessment (MBSA), CRT, CRT and Modified Water Swallowing Test (MWSA), Swallowing And Breath Sound Analysis, Clinical Swallow Examination (CSE) and CRT, and CSE alone. Based on gold standards, the prevalence of silent aspiration ranged from 5.6 to 53.3%, accounting for 42.9 to 69.8% of all aspiration events. More detailed characteristics are listed in Supplementary Table 2.

### 3.3 Quality assessment

Two reviewers utilized the QUADAS-2 to assess the methodological quality of the included studies independently (Table 1). Only 1 study without risk of bias and 4 studies without applicability concerns (11, 12, 15, 16). The main risk of bias came from flow and timing because not all patients were screened and patients did not receive the same gold standards (11, 16, 18–20).

**Table 1:** Methodological quality of the included studies.

Study	Screening tools	Risk of bias				Applicability concerns		
		Patient selection	Index test	Reference standard	Flow and timing	Patient selection	Index test	Reference standard
Ramsey et al. (11)	MBSA	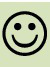	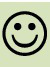	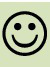	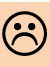	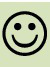	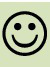	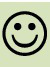
Wakasugi et al. (20)	CRT + MWSA	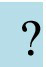	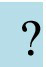	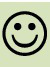	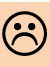	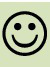	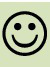	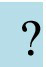
Kagaya et al. (16)	(a) CRT (First-step Simple Swallowing Provocation Test); (b) CRT (Second-step Simple Swallowing Provocation Test)	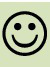	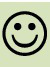	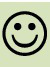	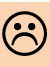	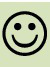	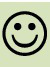	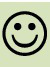
Sato et al. (12)	(a) CRT (30 s simple cough test); (b) CRT (60 s simple cough test)	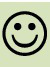	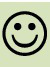	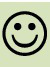	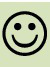	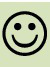	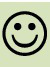	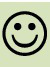
Shirazi et al. (18)	Swallowing and Breath Sound Analysis	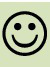	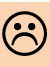	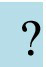	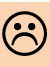	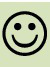	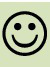	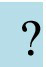
Lee et al. (17)	CRT	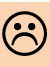	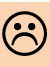	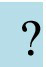	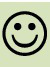	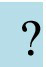	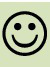	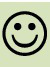
Wakasugi et al. (19)	CRT	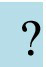	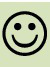	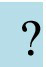	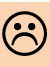	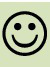	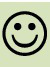	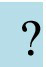
Guillén-Solà et al. (15)	CRT	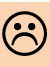	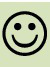	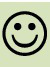	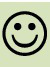	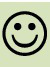	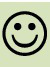	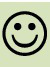
Trimble et al. (13)	CSE; (b) CRT; (c) CSE + CRT.	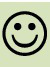	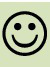	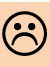	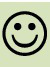	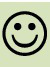	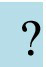	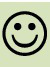

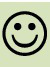
 Low Risk; 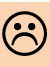 High Risk; 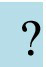 Unclear Risk.

MBSA, Modified Bedside Swallowing Assessment; MWSA, Modified Water Swallowing Test; CRT, Cough Reflex Test; CSE, Clinical Swallow Examination.

### 3.4 Narrative synthesis of screening tools for silent aspiration

Due to insufficient studies, we conducted a descriptive analysis of the 5 screening tools for silent aspiration. MBSA assessed the risk of aspiration after stroke by pulse oximetry monitoring and contrast swallowing (11). Because of the small number of silent aspiration cases and a low false positive rate, MBSA yielded extremely low sensitivity [0 (95% CI: 0–0.71)] and high specificity [0.98 (95% CI: 0.9–1.0)]. The sensitivity and specificity of the citric acid cough reflex test combined with the modified swallowing water test for the diagnosis of silent aspiration were 0.71 (95% CI: 0.53–0.85) and 0.88 (95% CI: 0.78–0.94) (20). Shirazi and colleagues analyzed 185 swallows from 11 patients using swallowing and breath sound analysis, demonstrating an accuracy, specificity, and sensitivity of 0.86 for diagnosing silent aspiration (18). Trimble’s study compared the diagnostic accuracy of CSE and the combination of CSE and CRT to screen silent aspiration (13). The sensitivity and specificity of CSE were 0.71 (95% CI: 0.29–0.96) and 0.88 (95% CI: 0.62–0.98), respectively, while the combination of CSE and CRT was 0.86 (95% CI: 0.42–1.0) and 0.69 (95% CI: 0.41–0.89) (Supplementary Figure 1).

### 3.5 Meta-analysis

Six studies compared the diagnostic accuracy of CRT and FEES/VFSS. Kagaya’s study involved two different doses of CRTs, while Sato’s study involved different induction times of CRTs (12, 16). Therefore, the 8 CRTs were enrolled in the meta-analysis. Supplementary Table 3 presents the methodologies and results interpretation of the 8 CRTs.

**Figure 2:**
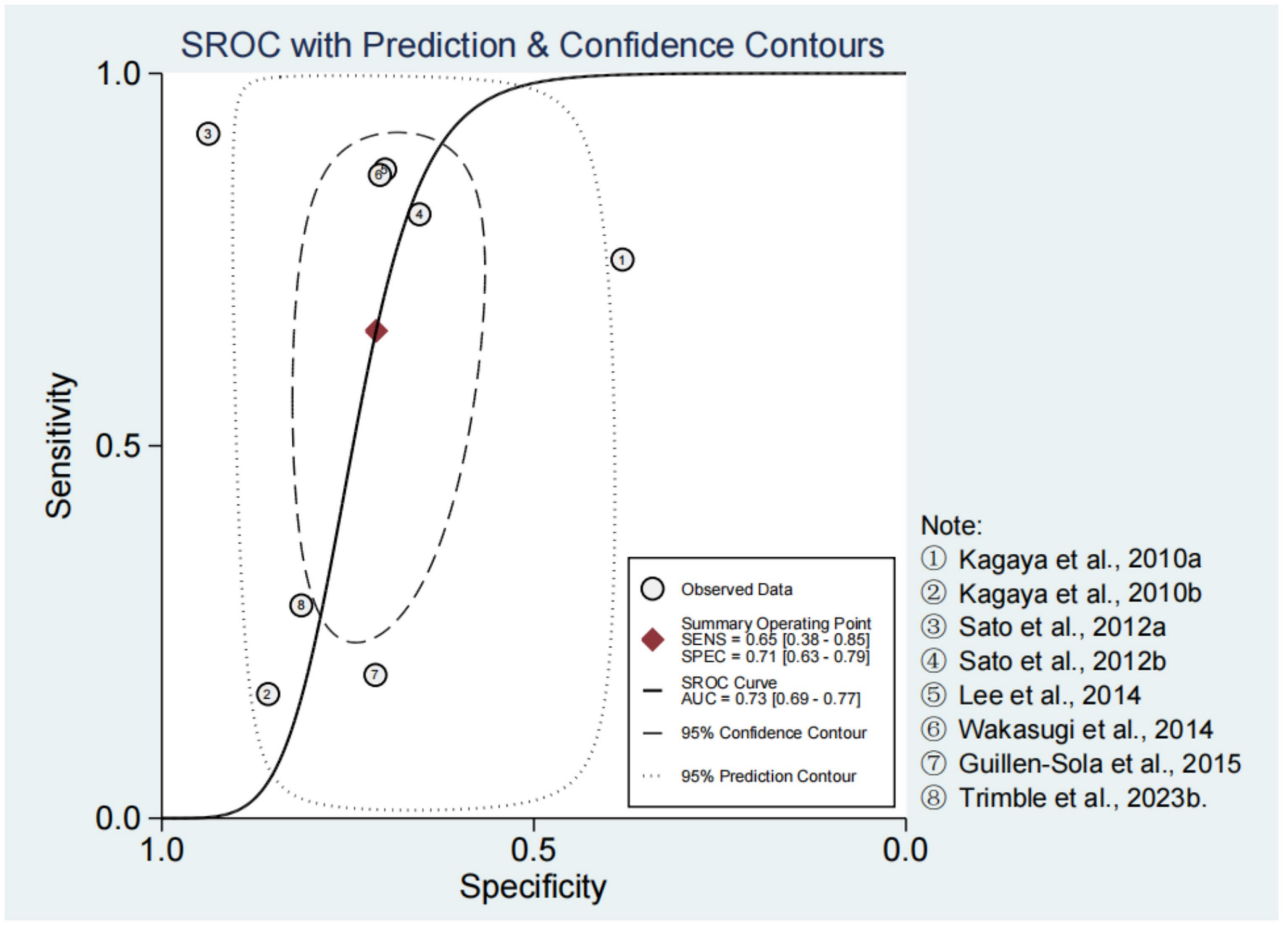
The SROC plot for CRT.

Spearman’s correlation coefficient and *p*-value were −0.10 and 0.82, respectively. In addition, there was no obvious “shoulder-arm” distribution observed in the SROC curve (Figure 2). These results suggested that there was no threshold effect in the included studies. In the bivariate boxplot, the First-step (0.4 mL) Simple Swallowing Provocation Test and the 30 Seconds Simple Cough Test were located outside the graph, indicating significant heterogeneity among the studies (12, 16) (Supplementary Figure 2).

**Figure 3:**
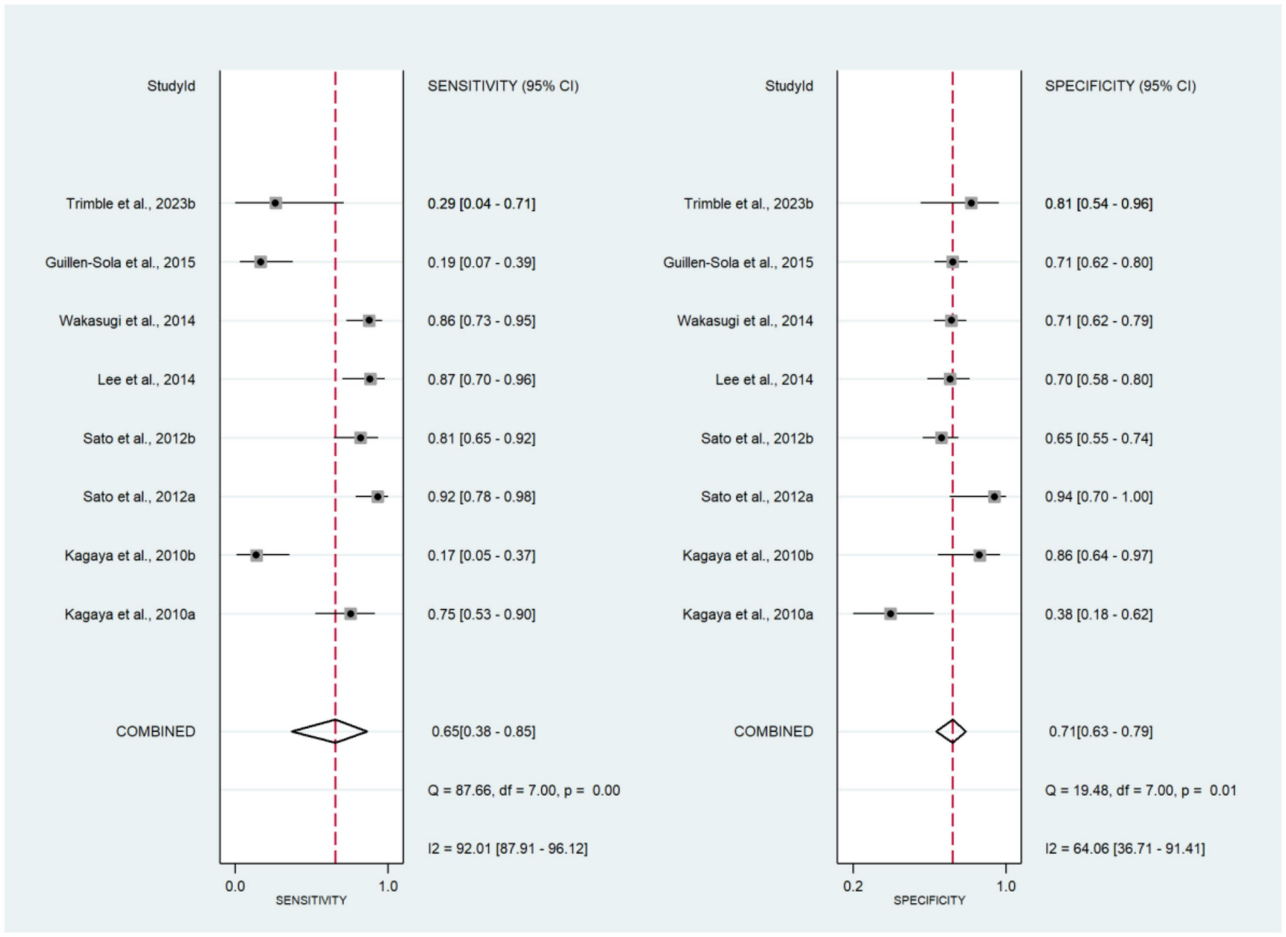
Forest plot of sensitivity and specificity for CRT.

The forest plots of sensitivity and specificity are shown in Figure 3. The combined sensitivity and specificity were 0.65 (95% CI: 0.38–0.85) and 0.71 (95% CI: 0.63–0.79), respectively. The PLR, NLR, and DOR were 2.27 (95% CI: 1.49–3.47), 0.49 (95% CI: 0.24–0.99), and 4.68 (95% CI: 1.57–13.98), respectively (Supplementary Figures 3, 4). In Figure 2, the AUC of CRT was 0.73 (95% CI: 0.69–0.77). The studies on the left side of the SROC are superior to those on the right. The studies closer to the upper left corner have higher diagnostic accuracy (Figure 2). The Fagan nomogram set the prior probability at 50%. The posterior probability was 87% when the CRT diagnosis was positive and 13% when the CRT diagnosis was negative (Supplementary Figure 5). The Deeks’ funnel plot indicated that no significant publication bias was found among the studies (*p* = 0.43) (Supplementary Figure 6).

### 3.6 Subgroup analysis

The subgroups are defined by sample size, gold standard, induction substance, and induction time in Table 2. There was relatively little difference in sensitivity (0.63 vs. 0.72) and specificity (0.73 vs. 0.69) between studies with sample sizes less than 100 and greater than 100. Six studies used VFSS as the gold standard and yielded moderate sensitivity of 0.66 (95% CI: 0.58–0.73) and specificity of 0.69 (95% CI: 0.64–0.74) after combination. Sensitivity and specificity were similar for induction time of 60 s and less than 60 s. The sensitivity (0.75 vs. 0.76) and specificity (0.71 vs. 0.62) of citric acid-physiological saline-induced CRT were higher than those of water-induced CRT.

**Table 2:** Subgroup analysis.

Category	*N*	Sensitivity	*P*_1_	Specificity	*P*_2_
Sample size					
≤100	4	0.63 (0.52–0.73)	0.00	0.73 (0.61–0.83)	0.00
>100	4	0.72 (0.64–0.80)	0.00	0.69 (0.65–0.74)	0.00
Gold standard					
VFSS	6	0.66 (0.58–0.73)	0.00	0.69 (0.64–0.74)	0.00
VFSS/FEES	1	0.86 (0.73–0.95)	-	0.71 (0.62–0.79)	-
FEES	1	0.29 (0.04–0.71)	-	0.81 (0.54–0.96)	-
Induction substance					
Water	2	0.46 (0.31–0.61)	0.00	0.62 (0.46–0.76)	0.00
Citric acid-physiological saline	6	0.75 (0.68–0.81)	0.00	0.71 (0.66–0.75)	0.17
Induction time					
<60s	4	0.63 (0.52–0.73)	0.00	0.73 (0.61–0.83)	0.00
60s	4	0.72 (0.64–0.80)	0.00	0.69 (0.65–0.74)	0.79

### 3.7 Sensitivity analysis

Sensitivity analysis was performed by eliminating individual studies one by one. The fluctuation ranges of DOR, sensitivity, specificity, and AUC were relatively small, which indicated that the results of the analysis were not overly dependent on any single study and the results of the meta-analysis were robust (Supplementary Table 4).

## 4 Discussion

Previous studies typically combined silent aspiration with overt aspiration to screen aspiration risk in patients with dysphagia (21, 22). This is the first time that silent aspiration has been addressed as an independent topic for meta-analysis, and the accuracy of six screening tools has been explored in this context. Although VFSS and FEES were used as gold standards across the studies, the limited number of studies made it difficult to conduct cross-sectional comparisons among the 5 screening tools analyzed descriptively. Therefore, this study only conducted a meta-analysis on the diagnostic accuracy of CRT compared to the gold standards and obtained some significant conclusions.

The higher incidence and lower attention contribute to a greater rate of missed diagnoses for silent aspiration in patients with dysphagia. Taking the gold standard as a reference, the prevalence of silent aspiration varied widely and accounted for 42.9 to 69.8% of all aspiration events. Recent studies have found that clinical swallowing assessments may overlook up to 50% of silent aspiration cases (23). This phenomenon indicates that specialized screening for silent aspiration is essential for patients with dysphagia. Many silent aspiration events can evolve into aspiration pneumonia imperceptibly, and the severity of the condition mainly depends on several factors, including the quantity and nature of aspirated material, the frequency of aspiration, and the host’s response to the aspirated substances (24, 25). Therefore, healthcare institutions should enhance awareness and focus on silent aspiration to reduce its incidence and related complications, ultimately providing comprehensive care and support for patients with dysphagia.

VFSS and FESS are currently the most commonly used diagnostic tools for detecting silent aspiration (9). However, both methods can only observe aspiration events that occur during the examination. VFSS utilizes X-ray imaging to dynamically record the swallowing process with contrast agents, clearly showing the timing of aspiration (before, during, or after swallowing) as well as anatomical abnormalities such as laryngeal penetration and vallecular residue (26). FESS allows for direct endoscopic observation of the laryngeal structures, enabling the identification of secretions or food remnants above or below the vocal cords (27). Despite their utility, both methods capture only the aspiration events that occur during the assessment, potentially missing intermittent incidents. Additionally, both procedures are invasive and cannot provide continuous monitoring over a 24-h period. Therefore, the effectiveness of these instrumental assessments in predicting the risk of silent aspiration and ensuring ongoing monitoring is limited.

Compared to instrumental examination, the Swallowing And Breath Sound Analysis is non-invasive and efficient (18). This study found that the Swallowing And Breath Sound Analysis exhibited good sensitivity and specificity among all screening tools. Patients were given different types of solid and liquid foods when using this screening tool. The breathing and swallowing sounds are separated through the recorded sound signals, and the sound frequency was analyzed to diagnose silent aspiration (28). However, the procedure is undoubtedly delicate and complex. Any abnormal sound can interfere with the final diagnostic results. Only one study analyzed the diagnostic value of Swallowing And Breath Sound Analysis. We cannot easily conclude that it is the most accurate screening tool for silent aspiration other than VFSS and FEES.

CRT exhibits moderate sensitivity and specificity and is the most widely used screening tool for silent aspiration. The CRT assesses laryngeal sensory function and cough reflex by inducing coughing using different doses of water or citric acid (16, 17). In subgroup analysis, the sensitivity (0.75 vs. 0.46) and specificity (0.71 vs. 0.62) of the citric acid-induced CRT were higher than those of the water-induced CRT. The citric acid-induced CRT is administered through a nebulizer mask, while the water-induced CRT is administered via a syringe containing 0.4 mL or 2 mL of water. If the induced substance fails to elicit an effective reflex cough, it suggests that the airway is unable to respond protectively to aspiration (29). In terms of safety, the citric acid-induced CRT is safer than water-induced CRT, because performing water-induced CRT when patients have an ineffective cough reflex may increase the risk of aspiration. Therefore, it is essential to consider both safety and effectiveness when selecting appropriate screening tools for silent aspiration.

The First-step (0.4 mL) Simple Swallowing Provocation Test and the 30 Seconds Simple Cough Test were found to have significant heterogeneity in the bivariate boxplot, but no threshold effect was generated (12, 16). The heterogeneity may be attributed to differences between induction substance and induction time. After the First-step (0.4 mL) Simple Swallowing Provocation Test was excluded by sensitivity analysis, the AUC was 0.72 (0.68–0.76). Similarly, when the 30 Seconds Simple Cough Test was excluded, the AUC was 0.70 (0.66–0.74). Nonetheless, both tools can effectively screen silent aspiration and provide new directions for aspiration prevention in patients with dysphagia.

Screening for silent aspiration plays a significant guiding role in the management of dysphagia. The screening results can guide the direction of instrumental assessments and assist healthcare professionals in medical determination. Once silent aspiration is confirmed, it can guide the implementation of effective rehabilitation therapies, such as dietary modifications, compensatory strategies, swallowing exercises, enhanced monitoring, and intensified oral care. In the future, establishing a precision management cycle integrating “screening, diagnosis, and intervention” may fundamentally improve patient outcomes and reduce the incidence of silent aspiration.

Several limitations should be acknowledged in this study. Firstly, only narrative analysis was performed for 5 screening tools, including MBSA, CRT and MWST, CSE and CRT, Swallowing And Breath Sound Analysis, and CSE. Secondly, owing to the difference in induction substance, we could not analyze the effects of substance concentration on the diagnostic value of CRT by subgroup analysis. Thirdly, two studies combined two tools to screen silent aspiration. However, the limited studies prevented us from conducting a network meta-analysis (13, 20). Finally, the importance of silent aspiration in patients with dysphagia should be emphasized. More validation studies are required to apply silent aspiration screening tools in clinical practice.

## 5 Conclusion

In summary, the VFSS and FEES remain the widely used gold standards for diagnosing silent aspiration. The MBSA, CRT, CRT and MWST, CSE and CRT, Swallowing And Breath Sound Analysis, and CSE are practical screening tools for silent aspiration in patients with dysphagia. However, more diagnostic studies are needed to validate the application value of different screening tools in clinical settings, which will provide more precise evidence for the popularization of silent aspiration screening tools.

## Data Availability

The original contributions presented in the study are included in the article/Supplementary material, further inquiries can be directed to the corresponding author.
