# Supplementary material for: Diagnostic accuracy of screening tools for silent aspiration in patients with dysphagia: a systematic review and meta-analysis
Source: Front Neurol. 2025 Sep 10;16:1576869. doi: 10.3389/fneur.2025.1576869 (PMC12457149; doi:10.3389/fneur.2025.1576869)

*Supplementary Materials*

**Supplementary Tables**

Supplementary Table 1. Search strategy 2

Supplementary Table 2. Study characteristics 6

Supplementary Table 3. Methodologies and results interpretation of 8 CRTs 9

Supplementary Table 4. Sensitivity analysis 12

**Supplementary Figures**

Supplementary Fig. 1. Forest plot of five comparisons 13

Supplementary Fig. 2. Bivariate boxplot 14

Supplementary Fig. 3. Forest plot of positive and negative likelihood ratios 15

Supplementary Fig. 4. Forest plot of diagnostic score and odds ratio 16

Supplementary Fig. 5. Fagan’s nomogram 17

Supplementary Fig. 6. Deeks' funnel plot 18

**Supplementary Table 1. Search strategy**

| **Database** | **No** | **Search strategy** |
| --- | --- | --- |
| PubMed | #1 | ("deglutition disorders"[Mesh]) OR "deglutition"[Mesh] OR dysphagia |
|  | #2 | (swallow*[Title/Abstract] OR deglutit*[Title/Abstract] OR dysphag*[Title/Abstract] OR pharyn*[Title/Abstract] OR oropharyn*[Title/Abstract]) AND (disturbance[Title/Abstract] OR disorder[Title/Abstract] OR difficult*[Title/Abstract] OR dysfunction*[Title/Abstract] OR impair*[Title/Abstract] OR condition*[Title/Abstract] OR abnormal*[Title/Abstract] OR damage*[Title/Abstract] OR injur*[Title/Abstract]) |
|  | #3 | #1 OR #2 |
|  | #4 | (((silent aspiration[Title/Abstract]) OR (micro-aspiration*[Title/Abstract])) OR (microaspiration*[Title/Abstract])) OR (invisible aspiration[Title/Abstract]) |
|  | #5 | (silent[Title/Abstract] OR invisible[Title/Abstract] OR micro[Title/Abstract]) AND ("Pneumonia, Aspiration"[Mesh] OR "Respiratory Aspiration"[Mesh]) |
|  | #6 | #4 OR #5 |
|  | #7 | ((((("Diagnosis"[Mesh] OR "Early Diagnosis"[Mesh]) OR "Mass Screening"[Mesh]) OR "Sensitivity and Specificity"[Mesh]) OR "False Positive Reactions"[Mesh]) OR "False Negative Reactions"[Mesh]) OR "Reference Values"[Mesh] |
|  | #8 | diagnos*[Title/Abstract] OR screen*[Title/Abstract] OR accurac*[Title/Abstract] OR probabilit*[Title/Abstract] OR predict*[Title/Abstract] OR roc[Title/Abstract] OR receiver operat* characteristic[Title/Abstract] OR auc[Title/Abstract] OR sensitivity[Title/Abstract] OR specificity[Title/Abstract] OR false positive[Title/Abstract] OR true positive[Title/Abstract] OR false negative[Title/Abstract] OR true negative[Title/Abstract] OR TPR[Title/Abstract] OR FPR[Title/Abstract] OR TNR[Title/Abstract] OR FNR[Title/Abstract] OR reference value*[Title/Abstract] OR likelihood ratio*[Title/Abstract] OR detect*[Title/Abstract] OR scale*[Title/Abstract] OR checklist*[Title/Abstract] OR assess*[Title/Abstract] OR exam*[Title/Abstract] OR identif*[Title/Abstract] OR recogni*[Title/Abstract] OR evaluat*[Title/Abstract] OR risk[Title/Abstract] OR test*[Title/Abstract] OR valid*[Title/Abstract] OR reliability[Title/Abstract] |
|  | #9 | #7 OR #8 |
|  | #10 | #3 AND #6 AND #9 |
| **Database** | **No** | **Search strategy** |
| Embase | #1 | 'dysphagia'/exp OR 'swallowing'/exp |
|  | #2 | (swallow*:ti,ab,kw OR deglutit*:ti,ab,kw OR dysphag*:ti,ab,kw OR pharyn*:ti,ab,kw OR oropharyn*:ti,ab,kw) AND (disturbance:ti,ab,kw OR disorder:ti,ab,kw OR difficult*:ti,ab,kw OR dysfunction*:ti,ab,kw OR impair*:ti,ab,kw OR condition*:ti,ab,kw OR abnormal*:ti,ab,kw OR damage*:ti,ab,kw OR injur*:ti,ab,kw) |
|  | #3 | #1 OR #2 |
|  | #4 | 'silent aspiration':ti,ab,kw OR microaspiration:ti,ab,kw OR 'micro aspiration':ti,ab,kw OR 'invisible aspiration':ti,ab,kw |
|  | #5 | (silent:ti,ab,kw OR invisible:ti,ab,kw OR micro:ti,ab,kw) AND ('aspiration'/exp OR 'aspiration pneumonia'/exp OR 'pulmonary aspiration'/exp) |
|  | #6 | #4 OR #5 |
|  | #7 | 'diagnostic procedure'/exp OR 'diagnosis'/exp OR 'screening'/exp OR 'sensitivity and specificity'/exp OR 'diagnostic error'/exp OR 'receiver operating characteristic'/exp |
|  | #8 | diagnos*:ti,ab,kw OR screen*:ti,ab,kw OR accurac*:ti,ab,kw OR probabilit*:ti,ab,kw OR predict*:ti,ab,kw OR roc:ti,ab,kw OR 'receiver operat* characteristic':ti,ab,kw OR sensitivity:ti,ab,kw OR specificity:ti,ab,kw OR 'false positive':ti,ab,kw OR 'true positive':ti,ab,kw OR 'false negative':ti,ab,kw OR 'true negative':ti,ab,kw OR tpr:ti,ab,kw OR fpr:ti,ab,kw OR tnr:ti,ab,kw OR fnr:ti,ab,kw OR 'reference value*':ti,ab,kw OR 'likelihood ratio*':ti,ab,kw OR detect*:ti,ab,kw OR scale*:ti,ab,kw OR checklist*:ti,ab,kw OR assess*:ti,ab,kw OR exam*:ti,ab,kw OR identif*:ti,ab,kw OR recogni*:ti,ab,kw OR evaluat*:ti,ab,kw OR risk:ti,ab,kw OR test*:ti,ab,kw OR valid*:ti,ab,kw OR reliability:ti,ab,kw |
|  | #9 | #7 OR #8 |
|  | #10 | #3 AND #6 AND #9 |
| **Database** | **No** | **Search strategy** |
| Cochrane Libaray | #1 | MeSH descriptor: [Deglutition] explode all trees |
|  | #2 | MeSH descriptor: [Deglutition Disorders] explode all trees |
|  | #3 | #1 OR #2 |
|  | #4 | (swallow* OR deglutit* OR dysphag* OR pharyn* OR oropharyn*):ti,ab,kw AND (disturbance OR disorder OR difficult* OR dysfunction* OR impair* OR condition* OR abnormal* OR damage* OR injur*):ti,ab,kw |
|  | #5 | #3 OR #4 |
|  | #6 | (silent aspiration):ti,ab,kw OR (microaspiration):ti,ab,kw OR (micro-aspiration):ti,ab,kw OR (invisible aspiration):ti,ab,kw |
|  | #7 | MeSH descriptor: [Pneumonia, Aspiration] explode all trees |
|  | #8 | MeSH descriptor: [Respiratory Aspiration] explode all trees |
|  | #9 | (silent):ti,ab,kw OR (invisible):ti,ab,kw OR (micro):ti,ab,kw |
|  | #10 | #6 OR(#9 AND (#7 OR #8)) |
|  | #11 | MeSH descriptor: [Early Diagnosis] explode all trees |
|  | #12 | MeSH descriptor: [Mass Screening] explode all trees |
|  | #13 | MeSH descriptor: [Sensitivity and Specificity] explode all trees |
|  | #14 | MeSH descriptor: [False Positive Reactions] explode all trees |
|  | #15 | MeSH descriptor: [False Negative Reactions] explode all trees |
|  | #16 | MeSH descriptor: [Reference Values] explode all trees |
|  | #17 | MeSH descriptor: [Diagnosis] explode all trees |
|  | #18 | #11 OR #12 OR #13 OR #14 OR #15 OR #16 OR #17 |
|  | #19 | (diagnos* OR screen* OR accurac* OR probabilit* OR predict* OR roc OR auc OR receiver operat* characteristic OR sensitivity OR specificity OR false positive OR true positive OR false negative OR true negative OR TPR OR FPR OR TNR OR FNR OR reference value* OR likelihood ratio* OR detect* OR scale* OR checklist* OR assess* OR exam* OR identif* OR recogni* OR evaluat* OR risk OR test* OR valid* OR reliability):ti,ab,kw |
|  | #20 | #18 OR #19 |
|  | #21 | #5 AND #10 AND #20 |
| **Database** | **No** | **Search strategy** |
| CINAHL | #1 | (MM "Deglutition") OR (MM "Deglutition Disorders") |
|  | #2 | TX ( swallow* OR deglutit* OR dysphag* OR pharyn* OR oropharyn* ) AND TX ( disturbance OR disorder OR difficult* OR dysfunction* OR impair* OR condition* OR abnormal* OR damage* OR injur* ) |
|  | #3 | #1 OR #2 |
|  | #4 | TX silent aspiration OR TX microaspiration* OR TX micro-aspiration* OR TX invisible aspiration |
|  | #5 | TX silent OR TX micro OR TX invisible |
|  | #6 | (MM "Aspiration") OR (MM "Pneumonia, Aspiration") OR (MM "Risk for Aspiration (NANDA)") OR (MM "Aspiration Risk (Saba CCC)") |
|  | #7 | #5 AND #6 |
|  | #8 | #4 OR #7 |
|  | #9 | (MH "Diagnosis+") OR (MH "Early Diagnosis+") OR (MM "Sensitivity and Specificity") OR (MM "False Positive Results") OR (MM "False Negative Results") OR (MH "Reference Values+") OR (MM "ROC Curve") |
|  | #10 | TX diagnos* OR screen* OR accurac* OR probabilit* OR predict* OR roc OR auc OR receiver operat* characteristic OR sensitivity OR specificity OR false positive OR true positive OR false negative OR true negative OR TPR OR FPR OR TNR OR FNR OR reference value* OR likelihood ratio* OR detect* OR scale* OR checklist* OR assess* OR exam* OR identif* OR recogni* OR evaluat* OR risk OR test* OR valid* OR reliability |
|  | #11 | #9 OR #10 |
|  | #12 | #3 AND #8 AND #11 |
| **Database** | **No** | **Search strategy** |
| Scopus | #1 | TITLE-ABS-KEY(Deglutition) OR TITLE-ABS-KEY-AUTH(dysphagia) OR TITLE-ABS-KEY-AUTH(Deglutition Disorders) |
|  | #2 | (TITLE-ABS-KEY(swallow* OR deglutit* OR dysphag* OR pharyn* OR oropharyn*) OR TITLE-ABS-KEY(disturbance OR disorder OR difficult* OR dysfunction* OR impair* OR condition* OR abnormal* OR damage* OR injur*)) |
|  | #3 | #1 OR #2 |
|  | #4 | (TITLE-ABS-KEY (silent AND aspiration) OR TITLE-ABS-KEY (micro-aspiration) OR TITLE-ABS-KEY (microaspiration) OR TITLE-ABS-KEY (invisible AND aspiration)) |
|  | #5 | ( TITLE-ABS-KEY ( silent OR micro OR invisible ) AND TITLE-ABS-KEY ( aspiration AND pneumonia OR aspiration OR pulmonary AND aspiration ) ) |
|  | #6 | #4 OR #5 |
|  | #7 | TITLE-ABS-KEY ( diagnos* OR screen* OR accurac* OR probabilit* OR predict* OR roc OR auc OR ( receiver AND operat* AND characteristic ) OR sensitivity OR specificity OR ( false AND positive ) OR ( true AND positive ) OR ( false AND negative ) OR ( true AND negative ) OR tpr OR fpr OR tnr OR fnr OR ( reference AND value* ) OR ( likelihood AND ratio* ) OR detect* OR scale* OR checklist* OR assess* OR exam* OR identif* OR recogni* OR evaluat* OR risk OR test* OR valid* OR reliability ) |
|  | #8 | #3 AND #6 AND #7 |
| **Database** | **No** | **Search strategy** |
| Web of Science | #1 | TS=(Deglutition OR Deglutition disorder OR dysphagia) |
|  | #2 | (TS=(swallow* OR deglutit* OR dysphag* OR pharyn* OR oropharyn*)) AND TS=(disturbance OR disorder OR difficult* OR dysfunction* OR impair* OR condition* OR abnormal* OR damage* OR injur*) |
|  | #3 | #1 OR #2 |
|  | #4 | (((TS=(silent aspiration)) OR TS=(micro-aspiration)) OR TS=(microaspiration)) OR TS=(invisible aspiration) |
|  | #5 | (TS=(silent OR invisible OR micro)) AND TS=((aspiration pneumonia) OR (pulmonary aspiration)) |
|  | #6 | #4 OR #5 |
|  | #7 | TS=(diagnos* OR screen* OR accurac* OR probabilit* OR predict* OR roc OR receiver operat* characteristic OR roc OR auc OR sensitivity OR specificity OR false positive OR true positive OR false negative OR true negative OR TPR OR FPR OR TNR OR FNR OR reference value* OR likelihood ratio* OR detect* OR scale* OR checklist* OR assess* OR exam* OR identif* OR recogni* OR evaluat* OR risk OR test* OR valid* OR reliability) |
|  | #8 | #3 AND #6 AND #7 |

**Supplementary Table 2.** Study characteristics

| Author,  Year (Country) | *N* | Age  (Mean±SD) | Disease | Screening tools | Gold standards | Aspiration substance | Prevalence of SA | Proportion of SA to all aspiration | TP | FP | FN | TN |
| --- | --- | --- | --- | --- | --- | --- | --- | --- | --- | --- | --- | --- |
| Ramsey, 2006 (England) | 54 | 70.9±10.2 | Acute stroke | Modified Bedside Swallowing Assessment | VFSS | Patients were given three 5-mL contrast agent, followed by 75 ml water. | 5.6% | 42.9% | 0 | 1 | 3 | 50 |
| Wakasugi, 2008 (Japan) | 107 | 69.9±11.7 | Dysphagia(cardiovascular disease; head or neck cancer; neuromuscular disease; respiratory disease; other diseases) | Cough reflex test+modified water swallowing test | VFSS/FEES | Patients inhaled a mist of citric acid-physiologic saline orally for 1 min | 31.8% | 68% | 24 | 9 | 10 | 64 |
| Kagaya, 2010a (Japan) | 45 | 76±12 | Dysphagia(aspiration pneumonia; Stroke; Surgery; Parkinson’s disease; osteophyte of the cervical spine) | CRT (First-step Simple Swallowing Provocation Test) | VFSS | 0.4 ml water | 53.3% | 44.4% | 18 | 13 | 6 | 8 |
| Kagaya, 2010b (Japan) | 45 | 76±12 | Dysphagia(aspiration pneumonia; stroke; surgery; Parkinson’s disease; osteophyte of the cervical spine) | CRT (Second-step Simple Swallowing Provocation Test) | VFSS | 2 ml water | 53.3% | 44.4% | 4 | 3 | 20 | 18 |
| Sato, 2012a (Japan) | 53 | 72±16 | Dysphagia(cerebrovascular disease; neuromuscular disease; respiratory disease; miscellaneous disease) | CRT (30 seconds simple cough test) | VFSS | Patients inhaled a mist of 1% w/v citric acid-physiological saline for 30 seconds. | 26.2% | 69.8% | 34 | 1 | 3 | 15 |
| Sato, 2012b (Japan) | 141 | 71±14 | Dysphagia(cerebrovascular disease; disuse syndrome; neuromuscular disease; respiratory disease; cancer; cervical spine injury;  miscellaneous disease) | CRT (60 seconds simple cough test) | VFSS | Patients inhaled a mist of 1% w/v citric acid-physiological saline for 1 min. | 26.2% | 69.8% | 30 | 36 | 7 | 68 |
| Shirazi, 2012 (Canada) | 11 | 58.4±18.2 | Dysphagia (stroke; neurodegenerative disorders; traumatic brain injury) | Swallowing and Breath Sound Analysis | VFSS/FEES | Solid and liquid food | 20% | - | 32 | 20 | 5 | 128 |
| Lee, 2014 (Korea) | 101 | 73.0±9.2 | Dysphagia (ischemic stroke; intracerebral hemorrhage; traumatic brain injury; encephalitis; hypoxic brain damage; Parkinson’s disease) | CRT | VFSS | Patients inhaled a mist of 1% w/v citric acid-physiological saline for 1 min. | 30.7% | 50.8% | 27 | 21 | 4 | 49 |
| Wakasugi, 2014 (Japan) | 160 | 70±13 | Dysphagia (cerebrovascular disease; neuromuscular disease; head and neck cancer; respiratory disease; other diseases) | CRT | VFSS/FEES | Patients inhaled a mist of 1% w/v citric acid-physiological saline for 1 min. | 27.5% | 55.7% | 38 | 34 | 6 | 82 |
| Guillen-Sola, 2015 (Spain) | 134 | 62.2±11.9 | Subacute Stroke | CRT | VFSS | Patients inhaled a mist of 1% w/v citric acid-physiological saline for 1 min. | 19.4% | - | 5 | 31 | 21 | 77 |
| Trimble, 2023a (America) | 23 | Men(65%): mean=72 Women(35%): range=54-89 | Hyperacute stroke | CSE | FEES | Patients were given fluids, semi-solid foods, and solid foods to induce coughing. | 30.4% | - | 5 | 2 | 2 | 14 |
| Trimble, 2023b (America) | 23 | Men(65%): mean=72 Women(35%): range=54-89 | Hyperacute stroke | CRT | FEES | Patients inhaled a mist of 0.6mol/L citric acid-physiological saline for 15 seconds. | 30.4% | - | 2 | 3 | 5 | 13 |
| Trimble, 2023c (America) | 23 | Men(65%): mean=72 Women(35%): range=54-89 | Hyperacute stroke | CSE+CRT | FEES | Oral diet and 0.6mol/L citric acid-physiological saline. | 30.4% | - | 6 | 5 | 1 | 11 |

Note: SA, silent aspiration; CRT, Cough Reflex Test; CSE, Clinical Swallow Examination; VFSS, videofluoroscopic swallowing study; FEES, flexible endoscopic evaluation of swallowing.

**Supplementary Table 3. Methodologies and results interpretation of 8 CRTs**

| Study | CRTs | Induction substance | Induction time | Method of application | Results interpretation |
| --- | --- | --- | --- | --- | --- |
| Kagaya et al., 2010a | First-step (0.4 ml) Simple Swallowing Provocation Test | water | 3s | SSPT was estimated by the latent time from the water injection to the onset of the swallowing reflex by visual observation following a bolus injection of 0.4 ml of distilled water at the suprapharynx through a small nasal catheter. | Silent aspiration was diagnosed when aspiration occurred without a cough response in less than 10 s. |
| Kagaya et al., 2010b | Second-step (2.0 ml) SSPT Simple Swallowing Provocation Test | water | 3s | SSPT was estimated by the latent time from the water injection to the onset of the swallowing reflex by visual observation following a bolus injection of 2.0 ml of distilled water at the suprapharynx through a small nasal catheter. | Silent aspiration was diagnosed when aspiration occurred without a cough response in less than 10 s. |
| Sato et al., 2012a | 30 Seconds Simple Cough Test | citric acid-physiological saline | 30s | Patients orally inhaled a mist of 1% w/v citric acid-physiological saline using a portable mesh nebulizer (NE-U22, Omrona). The nebulizer sprayed mist particles of approximately 5 micrometer size. Patients were asked to inhale deeply through the mouth according to verbal instruction and inhale citric acid several times until the first cough occurred. If patients had difficulty following these instructions, a nose clip was applied to encourage the patient to inhale orally. | No coughing within 1 minute of inhalation was considered negative |
| Sato et al., 2012b | 60 Seconds Simple Cough Test | citric acid-physiological saline | 60s | Patients orally inhaled a mist of 1% w/v citric acid-physiological saline using a portable mesh nebulizer (NE-U22, Omrona). The nebulizer sprayed mist particles of approximately 5 micrometer size. Patients were asked to inhale deeply through the mouth according to verbal instruction and inhale citric acid several times until the first cough occurred. If patients had difficulty following these instructions, a nose clip was applied to encourage the patient to inhale orally. | No coughing within 1 minute of inhalation was considered negative. |
| Lee et al., 2014 | Simplified Cough Test | citric acid-physiological saline | 60s | 1% citric acid-physiological saline solution from a commercially purchased portable nebulizer and mouth-piece. The citric acid mist was administered until the first cough. | Coughing within 28.12 seconds is considered negative or normal. |
| Wakasugi et al., 2014 | Cough Test | citric acid-physiological saline | 60s | Patients inhaled a mist of 1 w/v% citric acid-physiologic saline solution orally for 1 min with a hand-held nebulizer The examiner observed the number of times the patient coughed for 1 min during nebulizing. | More than five coughs were considered negative, while fewer than four were considered positive. |
| Guillen-Sola et al., 2015 | Citric acid cough test | citric acid-physiological saline | 60s | The citric acid cough test consists of oral inhalation of a 1.0 (weight by volume [w/v])% mixture of saline and citric acid for 1 minute through an ultrasonic nebulizer (OMRON NE-U17a) and the counting of cough peaks that result. The particle size was 1 to 8mm, and the output rate was 3mL/min. Patients were asked to breathe normally for 1 minute and cough as needed. | More than five coughs were considered negative, while fewer than four were considered positive. |
| Trimble et al., 2023b | Cough Reflex Test | citric acid-physiological saline | 15s | CRT was administered using a Microneb 3 jet nebulizer (Clement Clarke International, Essex, UK) with a restricted air flow rate of 6.6 L/min. A 0.6 mol/L citric acid solution diluted with 0.9% sodium chloride was used. | Positive was reported when participants produced fewer than two coughs on two of three trials |

**Supplementary Table 4. Sensitivity analysis**

| Study | DOR | Sensitivity | Specificity | AUC |
| --- | --- | --- | --- | --- |
| Kagaya et al., 2010a | 4.42(1.30-15.05) | 0.64(0.33-0.86) | 0.72(0.67-0.76) | 0.72(0.68-0.76) |
| Kagaya et al., 2010b | 6.00(2.12-16.99) | 0.73(0.49-0.88) | 0.69(0.65-0.73) | 0.70(0.65-0.74) |
| Sato et al., 2012a | 3.36(1.16-9.73) | 0.59(0.32-0.82) | 0.70(0.65-0.75) | 0.70(0.66-0.74) |
| Sato et al., 2012b | 4.52(1.26-16.26) | 0.62(0.32-0.85) | 0.73(0.62-0.82) | 0.75(0.71-0.78) |
| Lee et al., 2014 | 4.06(1.20-13.76) | 0.61(0.32-0.84) | 0.72(0.61-0.82) | 0.74(0.70-0.77) |
| Wakasugi et al., 2014 | 4.07(1.19-13.93) | 0.61(0.32-0.84) | 0.72(0.60-0.82) | 0.74(0.70-0.77) |
| Guillen-Sola et al., 2015 | 6.89(2.40-19.78) | 0.72(0.47-0.88) | 0.73(0.61-0.82) | 0.77(0.74-0.81) |
| Trimble et al., 2023b | 5.50(1.70-17.77) | 0.70(0.42-0.88) | 0.70(0.61-0.78) | 0.74(0.70-0.77) |

**Supplementary Fig. 1. Forest plot of five comparisons**


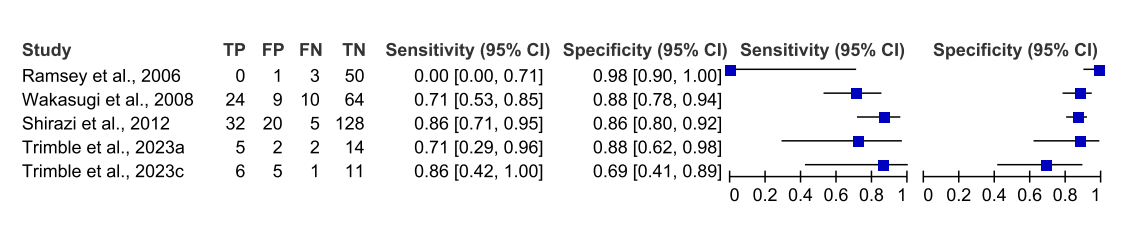


**Supplementary Fig. 2. Bivariate boxplot**


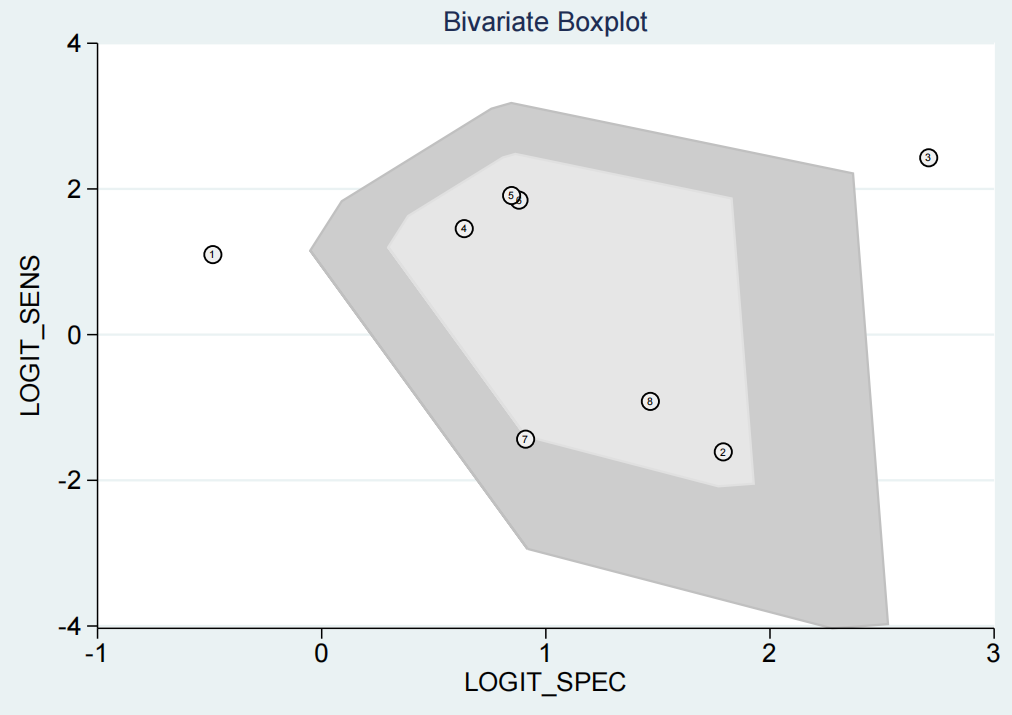


**Supplementary Fig. 3. Forest plot of positive and negative likelihood ratios**


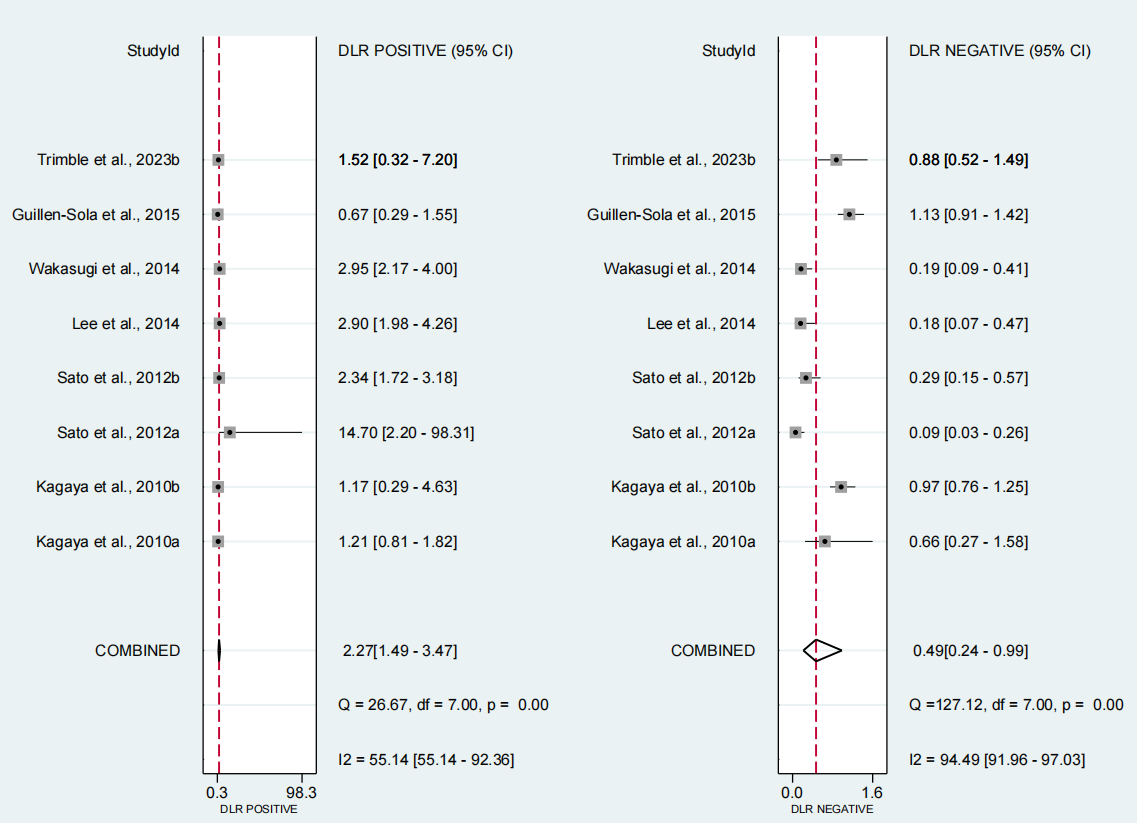


**Supplementary Fig. 4. Forest plot of diagnostic score and odds ratio**


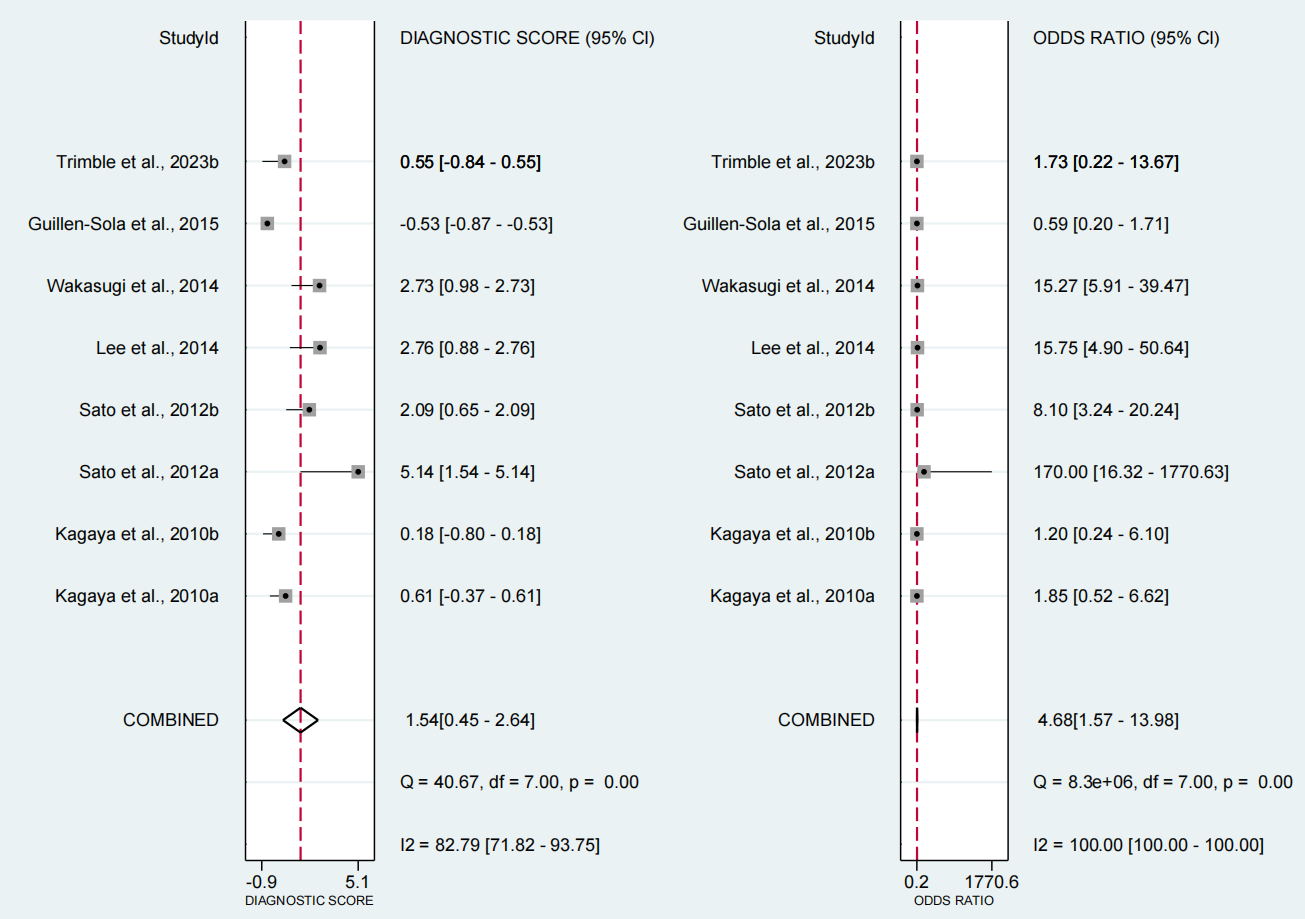


**Supplementary Fig. 5. Fagan’s plot**


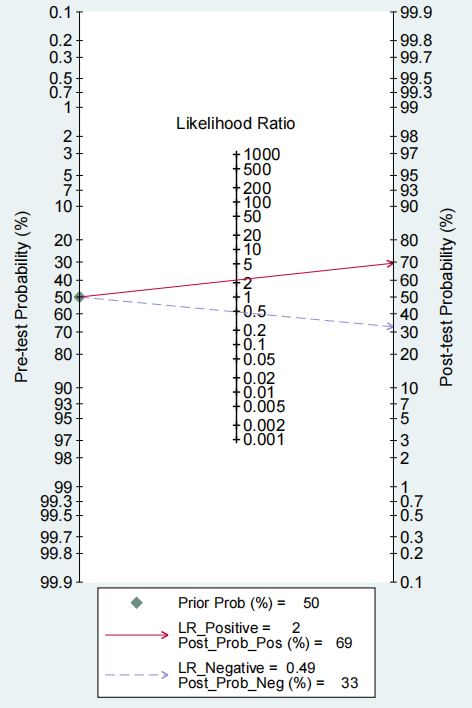


**Supplementary Fig. 6. Deek’s funnel plot**


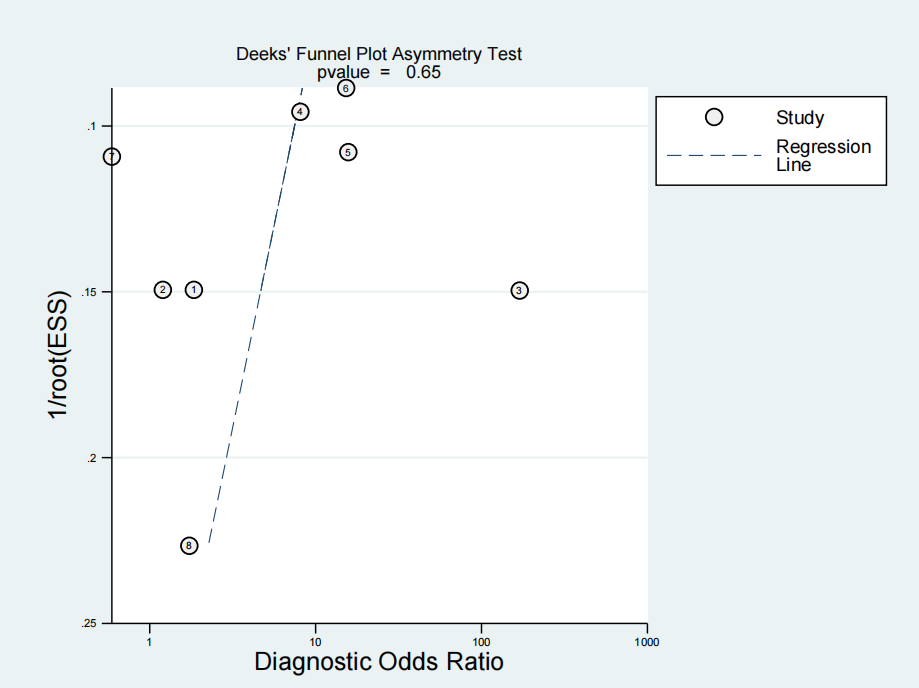

Supplement: Supplementary file 1 [file Supplementary_file_1.doc]
